# Search, save and share: family historians’ engagement practices with digital platforms

**DOI:** 10.1007/s10502-022-09404-4

**Published:** 2022-10-17

**Authors:** Henriette Roued, Helene Castenbrandt, Bárbara Ana Revuelta-Eugercios

**Affiliations:** 1 Department of Communication, University of Copenhagen, Copenhagen, Denmark; 2 Saxo Institute, University of Copenhagen, Copenhagen, Denmark; 3 Department of Economic History, Lund University, Lund, Sweden; 4 Danish National Archives, Odense, Denmark

**Keywords:** Family history, Digital platforms, Engagement, Serious leisure

## Abstract

Alongside established heritage institutions, family historians are central figures in the ecosystem of digital heritage, both as contributors to and users of digitized historical sources. With that in mind, this research aims for a wide examination of family historians' engagement with the broader selection of available digital platforms, providing knowledge about how and why they choose to use one platform over another. This knowledge is important for the future development of sustainable digital platforms in the heritage sector. With a large variety of digitized source providers, many with free access platforms, Denmark and Danish family historians make an excellent case for this study. Through both a questionnaire and focus group interviews, using a grounded theory approach, this study has developed a model of engagement with digital platforms, referred to as a buffet model. This model illustrates how family historians pick and choose from a selection of digital platforms throughout their search and management of information as well as their community interaction. Moreover, through the lens of the Serious Leisure Perspective we find that family history is often a life-long leisure activity and family historians’ usage of digital platforms support this finding.

## Introduction

There is an increasing focus on engagement and participation in archives and the role that community practitioners play in this (Benoit and Eveleigh 2019). For many archives, family historians are the largest user group and a long-term source of volunteer labor (Boyns 1999). A 2015 user study from the Danish National Archives confirms this. The archives’ services online (96%) as well as in the reading rooms (91%) are mainly used for the purpose of family history research (Tovgaard-Olsen 2015). Furthermore, family historians continue to be highly motivated contributors to the ongoing digitization and transcription of historical sources worldwide (Ridge 2017). As both contributors and users, family historians are a central figure in the ecosystem of digital heritage alongside established heritage institutions (Shaw and Donnelly 2021). As a practice, family history is key to the way millions of people engage with the past (Evans and de Groot 2019).

There has been research into family history as an activity, and in relation to identity and information behavior. However, there is still much more to learn about the interplay between family historians and the technologies that have emerged in recent decades. While archives may conduct internal user studies of their own digital services, this research aims for a wider examination of family historians' engagement with the broader selection of digital platforms available. This emphasizes the agency of family historians and is simply a question of knowing how and why they choose to use one platform over another. This knowledge is important for the future development of sustainable digital platforms in the heritage sector.

In order to do this, we focus on Danish family historians and how they find, use, manage, and share information online. This is an underexplored context despite the fact that family history in Denmark is carried out by a sizable and relatively socio-economically homogeneous community with good access to education and internet. There is furthermore a long tradition of using public records for family history research. Finally, the large variety of digitized source providers and a long history of free access makes Denmark an excellent case for this study.

The paper begins by giving a brief overview of previous research on family history, as well as the Danish digital platforms for family history research. Based on the idea of serious leisure, and with a grounded theory approach, we then present our buffet model to show how family historians engage with digital platforms. This model took shape as we analyzed the results from a questionnaire and focus group interviews, both carried out for this study.

### Family history

Family history as an activity has developed exponentially throughout the late twentieth and early twenty first centuries (Evans and de Groot 2019) and is thought to be one of the most popular activities online (Barnwell 2013). Hence, family history provides good opportunities to understand developments in online behavior (Veale 2009) because of practitioners’ avid practice of information seeking and sharing (Fulton 2009a).

Over the years family historians and their activities have been a field of interest to scholars, viewed from an institutional perspective, where family historians are an ever-growing user group, as well as from a sociological point of view, focusing on family history as a cultural phenomenon (Yakel and Torres 2007). Even so, several researchers point to the surprising fact that despite the popularity of family history and their central role in the ecosystem of digital heritage, little research has been published about this group (Boyns 1999; Barnwell 2013; Evans 2020b).

Current research on family historians can be found in small pockets across and between fields such as sociology (Bottero 2015), archival science (Niu 2021), information science (Friday 2014), genetics (Strand and Källén 2021), anthropology (Cannell 2011), digital humanities (Hoeve 2018), and public history (Shaw 2020). In this paper we are continuing in the footsteps of archival and information science as well as sociological scholarship, while using a digital heritage approach to focus on what family history means for our understanding of heritage data, platforms, and institutions.

There are past accounts of librarians and archivists being contemptuous toward family historians for their supposed ineptness at historical research and uncritical interpretation of records (Bidlack 1983). However, it is difficult to gauge the current acceptance or disdain amongst heritage professionals as this often comes up in informal conversation and is rarely documented (Evans 2020b). At most, we can perhaps reflect on institutional strategies focusing on “new” rather than “existing” users. However, family history has become a prosperous and growing industry with the potential to enable “a diverse group of people to experience a relationship with history” (Evans and de Groot 2019, (1), thus making family historians central figures in the heritage sector. In fact, Mckay (2002) reasons that family historians might well be influential allies for archival institutions, both politically and financially. Neither the political nor the fiscal climate for cultural institutions seem to have improved much in the 20 years since. In 2003 Duff and Johnson suggested that knowledge of family history research can be used to improve the design of archival information systems (Duff & Johnson, 2003). In the field of digital humanities Hoeve (2018) even argues for recognizing family historians as partners and team members in the cross-disciplinary use of digital technology. In that sense, many archivists and institutions have come to acknowledge the hobby of family history as a growing interest deserving specific attention, at least as the single largest user group in the reading rooms and online (Tovgaard-Olsen 2015). In general, the archival community has leveraged digital technologies, aiming to engage users and expand access to collections, in activities broadly covered by the umbrella term participatory archives (Benoit and Eveleigh 2019). These activities have been of great benefit to family historians who often take part enthusiastically (Ridge 2017). This paper provides a step toward a deeper understanding of family history and the role of digital platforms in the life cycle of this often long-running hobby.

Much of the previous research into family history has had an American focal point, likely linked to the fact that family history has long been a popular hobby in North America (Bidlack 1983). This is possibly fueled by the history of immigration and a wish to preserve memories of origin (Jurczyk-Romanowska and Tufekcic 2018). Even though its most common representations are closely tied to the Anglophone and Eurocentric constructs of sources and archives, family history is in essence a global phenomenon. Research into family history outside the above-mentioned contexts, as well as challenging the Eurocentric idea of kinship and history, is less available (Evans 2020a). Thus, we would like to acknowledge that while this paper studies a case of non-Anglophone family history, Danish source material still has origins in both Christian and colonial heritage and recordkeeping. This invariably influences the way we understand family history as an activity and is why we would encourage many more of these types of studies in different cultural contexts.

### Family history as information-seeking behavior

Family history research is inherently an information seeking activity. Through interviews with family historians, Duff and Johnston (2003) found that the activity can be divided into three iterative stages: (1) Collecting names of family members; (2) Collecting more detailed information such as dates, events, and place names (3) Collecting information about the historical and local context family members were part of. Yakel (2004) connects this last stage to the concept of orienting information (Savolainen 1995) which again is linked to the seeking of meaning and identity. With these stages comes several different strategies that were put into use when searching for new information. If a source or strategy failed the participants had no problem switching to another strategy (Duff and Johnston 2003). In a later study Friday (2014) proposes a cyclical and continuous model of search behavior. This model zooms in on the activities surrounding the actual search and in doing so presents some of the repetitive behaviors that form part of this activity. Moreover, Duff and Johnston (2003) found that in their information seeking family historians relied heavily on strong informal networks rather than on archivists or archival systems. The participants in their study did not think that archival search systems met their needs, as they were often provenance-based (i.e., structured according to the authority or institution that originally created the source) which is at odds with the information that family historians seek. Furthermore, their limited access to physical archives due to travel time and limited opening hours was an issue. Thus, they resorted to their own networks to help them overcome these barriers (Duff and Johnston, 2003). These social networks have their own culture and values of reciprocal information sharing which can be seen both offline and online (Yakel 2004; Fulton 2009a). However, as with institutional crowdsourcing projects (Zeeland and Gronemann 2019) most requests for aid are answered by a small group of “super-sharers” (Fulton 2009a; Willever-Farr et al 2012).

### Family history as serious leisure

In trying to understand the role of data and platforms in the information behavior of family historians we have, as in previous studies (Fulton 2009a; Friday 2014; Hershkovitz and Hardof-Jaffe 2017), turned to the concept of serious leisure. The Serious Leisure Perspective (SLP) was developed by Roberts Stebbins as he started his research on leisure in the 1970s. He recognizes three different types of leisure: casual, project-based, and serious (Stebbins 2009). Casual leisure is defined as a short-lived, intrinsically rewarding, and pleasurable activity that requires no special training (e.g., watching a movie or playing a board game). Project-based leisure is a short-term reasonably complicated undertaking that is done a single time or on sporadic occasions (e.g., volunteering for an event). In contrast to the first two, serious leisure is when people engage in activities that they find deeply fulfilling and that acquire special skills, knowledge and expertise, typically resulting in what can be called a leisure career. Serious leisure is also distinctive by requiring a great deal of personal effort as well as the “occasional need to persevere,” meaning that it is an activity that is not always just positive but sticking to it generally results in positive feelings. There are also several durable benefits, including self-enrichment, feeling of accomplishment, social interaction, and a sense of belongingness. Participants of serious leisure also develop a community spirit with shared attitudes, practices, values, etc., and they tend to strongly identify with their activity (Stebbins 2012). In our aim to develop a model of family history processes and the usage of digital platforms, serious leisure provides a further steppingstone to discuss the iterations of family history research. Understanding these processes as a part of a life-long serious leisure career also adds important perspectives to how we understand and design archival digital platforms.

### Danish digital platforms

Most archival institutions in Denmark offer varying degrees of access to their collections while also individuals, companies, and other organizations make resources available for family history. We distinguish between four categories of digital platforms: (1) publicly funded platforms; (2) nonprofit platforms; (3) for-profit platforms; (4) social media platforms. The resources available have increased especially during the last decade through ongoing digitization and transcription of historical sources. Through various collaborations, many of the same historical sources can also be accessed through different platforms.

Firstly, Danish national, municipal, and local archives have large amounts of digitized and transcribed collections freely available online through publicly funded platforms. The Danish National Archives has a large collection of digitized historical sources with more than 75 million scans of historical sources and 21 million transcribed census records freely available online. They began a digitization project as early as in 2002 with the site “Arkivalieronline” (translated: archival records online) that focused on two of the collections most used by family historians (i.e., censuses and parish records). The transcription project that predated this development began in the late 1990s as a collaborative project between family historians, researchers, and the archive which continues to this day (Rigsarkivet 2022a). This development is mirrored in similar projects by municipal and local archives.

Secondly, the international Family Search, an American non-profit stemming from the Church of Jesus Christ of Latter-day Saints (Mayfield 1983), has made billions of both digitized and indexed records available. Another platform is Danish Family Search. It is operated by two Danes residing in Australia as an alternative platform to access and transcribe Danish historical sources. Through collaborations with the National Archives, they provide a different interface to search both digitized and transcribed census and parish records. The site includes an interface for transcribing and linking these sources and the results of this are fed back to the National Archives. National organizations such as “Danske Slægtsforskere” (translated: Danish Family Historians) and “Samfundet for Dansk Genealogi og Personalhistorie” (translated: Society for Danish Genealogy and Personal History) offer a variety of resources including tutorials, a forum, and organizing various collaborative transcription projects. In addition, it is important to mention the various smaller societies, local volunteer-driven archives, and individuals who provide a wide variety of platforms with digitization and transcription of historical sources and different tools for accessing and understanding these sources.

Thirdly, Ancestry is the world’s largest for-profit family history platform, and was launched in 1996. To this date the site does not have a Danish version and Danish records first became accessible through the Swedish version in 2020 (Rigsarkivet 2022b). Nevertheless, the Ancestry platform does provide access to records outside of Denmark and the opportunity to upload and organize one's own family tree. In contrast, MyHeritage, founded in 2003 in Israel, has a better foothold in Denmark. Like Ancestry they offer a platform for organizing family trees and sharing them in a network while also automatically connecting family members using their large datasets as well as DNA testing.

Finally, the main family history resource on social media is the group “Slægtsforskning” (translated: Family History) on Facebook, with more than 25,000 members in 2022. It was established in 2007 by the national family history organization and can be seen as a continuation of their own forum from 2006. The forum and the Facebook group provide different ways to communicate with other family historians. While their daily activity level differs, it is still clear that asking questions and providing answers is a large part of the activity on both platforms. There are many other Facebook groups related to Danish family history. Some are on specific topics such as DNA and others are more regionally based groups. However, Danish family history activities seem to be much more limited on other social media platforms.

### Methodology

Our analysis drew on the concept of serious leisure and existing models of family history information behavior (see above), as well as a pilot project conducted by Revuelta-Eugercios. We have employed a constructivist grounded theory approach (Thornberg and Dunne 2019) where we continually collected and analyzed empirical material while continually and critically reflecting on existing literature (Charmaz 2014, 306) to further understand and develop a theory of the phenomenon in question. In this, we have been guided by the question: How do family historians engage with digital platforms? Specifically, which digital platforms do they use; how and why? As well as which opportunities and issues do these digital platforms present?

Through a questionnaire we gathered quantitative data on the platforms used, issues and advantages experienced online, and their information behavior. This enabled cross referencing with various demographic and experience related variables. Based on a preliminary analysis of these results we conducted a series of focus group interviews for a more qualitative understanding of the results. This continual analysis resulted in the development of a buffet model illustrating family historians' long-running engagement with digital platforms over many years.

### Questionnaire

In November 2020, we distributed a questionnaire, using the online SurveyXact platform, through some of the more popular Danish family history Facebook groups and through our contacts at the archives. Our aim was a wide distribution among family historians in Denmark. The questionnaire was distributed for one month and resulted in a total of 539 complete replies. The distribution of respondents across the regions of Denmark is similar to that of the Danish population (according to Statistics Denmark, dst.dk). The age distribution forms a bell-curve centered around the age group 65–74 and nearly two thirds of the respondents were women. When asked about the types of fields they had worked. Each respondent could choose more than one field if relevant and the definition of work was not limited to paid employment. Despite a wide distribution, the top three fields were (1) office work and accounting (22%); (2) health and care work (21%); (3) IT and technology (17%). Fewer responded that they had worked in manual labor fields as well as fields connected to cultural or religious institutions. While we did not ask about their educational or socio-economic backgrounds, we can conclude that the respondents generally represent a part of the population with a degree of higher education and experience with computer-based work. However, only few of the respondents had any connection to the fields from which the historical sources and digital platforms originate.

### Focus groups

All in all, 21 people took part across six focus group interviews. The interviews were conducted in Danish during January and February 2021 on the Zoom platform. Quotes from the interviews, used in the analysis, are translated by the authors. The participants were recruited among the respondents to the questionnaire and grouped according to which of four digital platforms they used for family history activities: (1) the National Archives as an example of a public platform; (2) Danish Family Search as an example of a non-profit platform; (3) MyHeritage as an example of a for-profit platform; (4) the group “Slægtsforskning” (translated: Family History) on Facebook, as an example of social media. Perhaps due to the Covid-19 restrictions, none of the participants seemed to question their participation in a virtual interview, even if this technology was new to some of them. This provided an unprecedented opportunity to gather people independent of their and our physical location and provided an easy way of recording the interview for further analysis alongside the interviewers’ notes.

Each interview began with a conversation about the participants' family history experiences and the geographical reach of their research. After this we moved into questions themed on one of the four platforms. The sessions ended with a question about privacy and data protection and how they cope with this. The final question provided a way to calibrate the overall atmosphere that had developed in the group. Whether it was one of positivity or negativity, a critical or an optimistic sentiment, which opinions dominated, and where they had originated. The interviews formed the basis for exemplifying and elaborating on the results from the questionnaire. Relevant quotes were coded in terms of their place in the model as it developed as a part of the grounded theory approach.

### Model of engagement with digital platforms

**Fig. 1:**
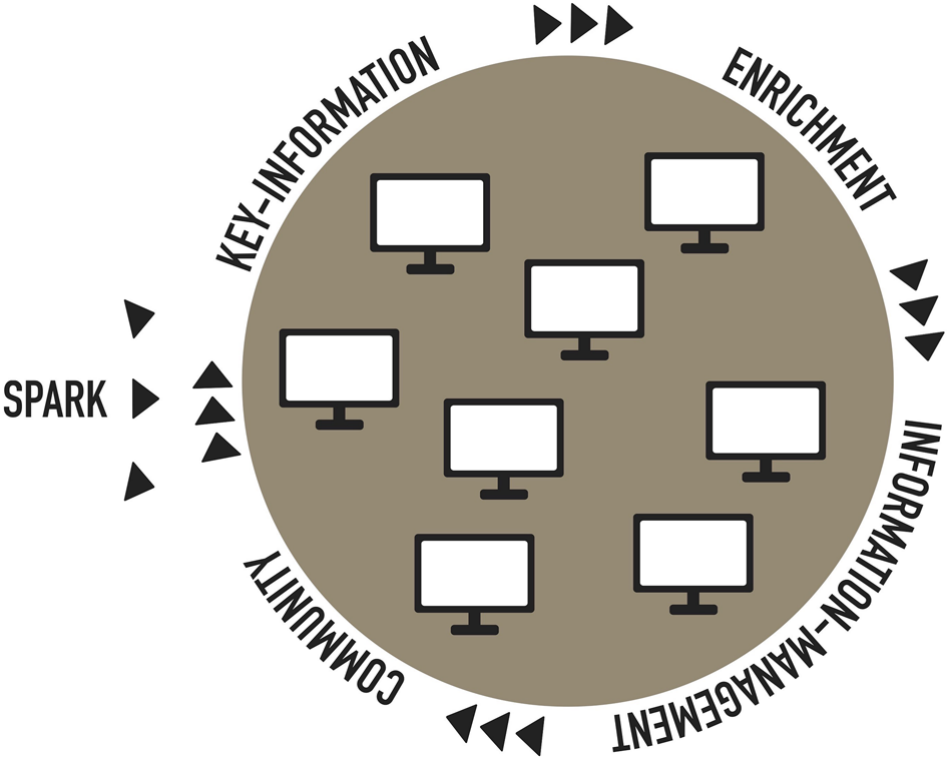
A buffet model of family historians’ engagement with digital platforms

To explain and illustrate the ways family historians engage with digital platforms we developed a buffet model (Fig. 1). Throughout the study we observed a cyclical pattern of engagement with digital platforms in the shape of information seeking as well as information management and community participation. Similar forms of cyclical patterns have been identified in previous studies of family history (Duff and Johnson 2003; Friday 2014) and were also observed in the pilot study. Using a grounded theory approach, we have analyzed the empirical data from the questionnaire and focus group interviews. Our analyses show how family historians use a wide variety of online resources, picking and choosing the best resource for each phase of their research as if it was a buffet. This is central to the model as the family historians are engaging with a buffet of platforms as they go through each phase of family history research in a cyclical manner.

Our model illustrates how family history engagement begins with a spark. This spark is commonly caused by some external or internal event which launches a process where four phases can be identified. All of these phases are to some extent present in any family history project but can appear both sequentially, and simultaneously within the other phases. The first phase is the collection of necessary *key-information*, typically in sources with high coverage like administrative records, parish records, and censuses. From this family historians often build what they call the skeleton of their family tree. A second phase is the *enrichment* of the basic information with other more focused historical sources covering different aspects of the historical person’s life. This is sometimes referred to as the meat on the bones (Jelks and Sikes 1983). These two phases normally happen in sequential order where the next two phases can also happen simultaneously. The third phase is *information management* which involves the process of collecting and preserving the information obtained. For instance, by sharing genealogies online, using family history software, creating family books, or just storing the information on a digital or analogue medium. A fourth phase involves participating in the wider *community* of family historians in some way. This could be participation in a family history group off- or online by sharing information and asking or answering questions. This could also be through family history related volunteering activities like teaching or supporting beginner family historians or by transcribing historical sources as a part of a crowdsourcing project.

After moving through this model additional sparks, triggered perhaps in some of the previous phases, launch family historians into new iterations of family history research. The conceptualization of multiple sparks allows the model to accommodate both family historians with a more project driven approach with clearly defined outcomes of work (one sequence) as well as those engaging in a serious leisure career. In the latter, the end of one cycle can, in general, not be distinguished from the beginning of a new one. Central in this model is a focus on how family historians engage with multiple digital platforms as they move through these phases. Family historians’ approach this as a buffet where they choose how, what, and when they engage with the different public, nonprofit, for-profit, and social media platforms. In the following sections, through our empirical material, we explore the platforms that family historians use, as well as the different phases in our model.

### The buffet: use of online platforms

Central to the buffet model are the digital platforms available to family historians. In this study, we have found that family historians use a variety of different platforms. In the questionnaire, respondents were asked to name up to ten websites they used the most in descending order. These free-text answers were then cleaned so that, for example, the 24 different ways of writing Ancestry (e.g., ancestry.com and ancestry.se) were labeled as one. These labels were then grouped into seven categories: (1) Danish National Archives, (2) Sall data, (3) Danish Family Search, (4) Family Search, (5) MyHeritage, (6) Ancestry, and (7) Other. The category “Other” is the most diverse and consists of over 100 different private, association, and archival platforms in and outside of Denmark. This question gave us an invaluable insight into the immense variety of platforms used by Danish family historians. While 94% of the respondents added at least one platform and almost half added up to four additional platforms only 11% added up to ten platforms. To get a sense of a platform's importance for the respondent we added a weighted count so that a platform mentioned first got a weight of 10 and a platform mentioned as the tenth got a weight of 1. A weighted sum was then calculated for each of the seven platform categories (Table 1).

**Table 1:** Seven platform categories with percentage of the weighted sum (adds up to 100%) and the percentage of respondents (*n* = 539) that mentioned the platforms in this category at least once

Platform category	Weighted sum	Respondents
Danish National Archives	41%	80%
Sall data	8%	30%
Danish Family Search	12%	45%
Family Search	9%	45%
MyHeritage	8%	30%
Ancestry	2%	15%
Other	20%	72%

Table 1 shows that Danish family historians’ use of platforms is varied and diverse. The single most used platform category (41% of the weighted sum and used by 80% of the respondents) covers the Danish National Archives’ various platforms such as the online catalog and the scans and transcriptions of parish and census records. Individual platforms (Sall data, Danish Family Search, Family History, MyHeritage, Ancestry) each make up a smaller part of the overall weighted sum and are mentioned by a smaller percentage of the respondents. The large and varied group of platforms in the category “Other” account for 20% of the weighted sum and 72% of the respondents mention one or more of the platforms in this category.

Several focus group participants specifically expressed this eclectic use of digital platforms as part of their family history experience. While talking about how to search for information one participant said: “At first, I try if I can figure out exactly where and when to look in parish records and censuses. Otherwise, I will try to search it all […] all the places I come to think of.” A very experienced family historian pointed out that to get access to all possible transcriptions of census records you had to search across at least four different platforms. Another participant termed family history as a type of detective work and pointed out that it is the different ways of finding information and the difficulties that it poses that makes it interesting: “It is really just about using your imagination […] You dig, and you find something, and it is really great when you find something. But it can take a long time, though. You need to take detours sometimes resulting in a dead end and then you need to start all over again. I find this exciting and without it would not be family history.” While pondering over how convenient it would be with one comprehensive platform that had it all, one participant concluded: “family history would probably also be a bit boring if one could just go in and, voila, there it is.”

This is echoed in other studies that have highlighted the importance of doing the work and the pleasure of discovery for the individual family historian (Lambert 1996). Bottero (2015) found that for the family historians she interviewed, their narrative of discovery existed alongside the stories they discovered. Thus, family history is not about knowing your family but rather a both pleasurable and painstaking journey (Fulton 2009b) of discovery through a buffet of different platforms. The many options for information discovery are not a problem to be solved, as some archivists may believe (Boyns 1999), but rather a core element in family history practices and the detective-like identity of family historians.

### Entering the model: sparks and iterations

A spark in this case is an internal or external event that launches the different phases and interaction with the buffet of platforms. In our focus groups participants often recounted how family events led them to a family history project. These events are not seldom of a tragic nature such as the death of older family members. One participant recalls that they “began in 2008 perhaps as many do first in that moment when I was clearing out after my parents. You resent the day you must do this but that does not change the fact. But there I found some papers and started digging into those which slowly got my research started.” These events can also be joyous and relate to the celebration of a family member. For instance, one participant began working on a new part of the family tree, in relation to the birth of a grandchild.

In the questionnaire, when we asked about potential initiators or sparks of family history activity half of the respondents referred to finding new sources of information about the family as a reason for engaging in family history. However, one important underlying factor was to have enough disposable time. Over 90% of the respondents gauged that they, on average, work on their family history every month and over half of them every week. Half of the respondents said that they do more family history during wintertime, although this is slightly more common among the older respondents and among women. However, only a quarter of the respondents did more family history when they were out of the job market (e.g., retired, students, unemployed). In fact, replies in the questionnaire add to the understanding of family history as a life-long activity (Hershkovitz and Hardof-Jaffe 2017). While some respondents can be classified as beginners (7% have been doing family history for up to 2 years), nearly 60% of the respondents have been doing family history for more than 10 years. While experience is generally related to age there are also younger respondents with several years of experience and older respondents with less experience. Even for the oldest groups, over 30 years of experience, gives them a starting point in mid-life. Consequently, according to the questionnaire family history seems to be more of a life-long hobby, something many begin in their young and mid adult lives, rather than a short-term project taken up in retirement.

Even so, there is reason to believe that family history had begun as a one-time activity or as a discrete project and developed into a longer engagement. In fact, one participant reported: “I was lured in by [a for-profit company] which said that you can do this in half a morning. It did start really well but it is a system where you quickly gain a large family, and it took time to sort that out.” In other words, his spark was advertising, aimed at his age group, promising fast results. Nevertheless, after this first discovery he also became hooked on finding original sources and discovering more on his own.

### The core phase: finding key-information and enriching it

**Fig. 2:**
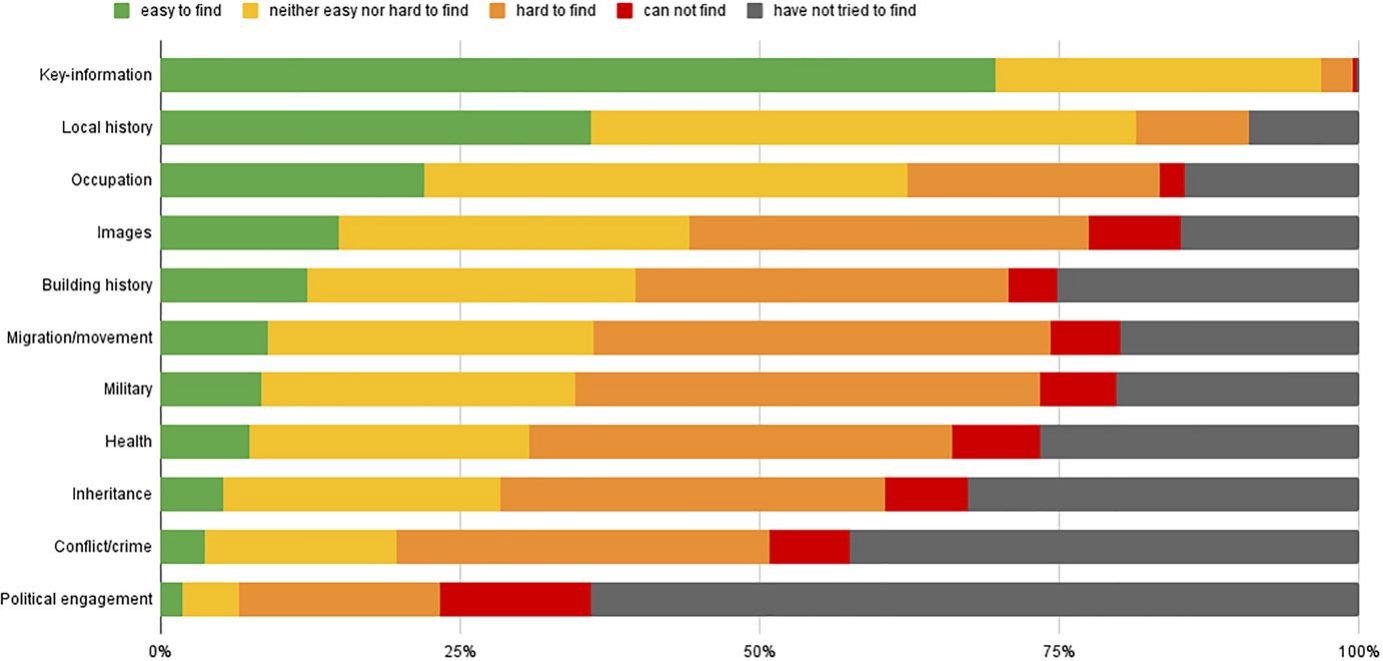
Diagram illustrating the questionnaire responses to the question: which types of information have you found online and how easy was it? Answer categories were: (1) easy to find, (2) neither easy nor hard to find, (3) hard to find, (4) cannot find, (5) have not tried to find. The information types were, in order of ease: key-information, local history, occupation, images, building history, migration/movement, military, disease, inheritance, conflict/crime, political engagement

After the initial spark family historians typically move into the phases of information searching using the buffet of platforms available. Most family historians begin by searching for key-information such as births, marriages, deaths, and family relationships. This is thought to be the easiest information to find online according to the respondents of the questionnaire (Fig. 2). Practically no one replied that they could not or had not tried to find this type of information and 70% gathered such information easily. Experience played a clear role here in that more experienced family historians said that this key-information was easy to find. This is in stark contrast to other types of sources such as information on local history (only 36% found this easy). Many other types of information such as health, migration/movement, or conflict/crime are generally more difficult to find, or the respondents have not even tried to find them for one reason or another. This is the case with political engagement according to approximately two thirds of the respondents. Here again it seems that experience plays a part where more experienced family historians are more likely to try to find such enriching information and overall find them easier.

This highlights that historical sources that provide key-information are both more sought after and easier to find. In a Danish context these include parish and census records. It is not surprising as such that the last 25 years of digitization and transcription have provided increasingly better access to precisely these types of sources across various platforms. The improved availability has also led to the boundaries between different institutions being fluent with less significance placed on where searchers are made, or as one respondent put it “I do not limit myself to the Danish National Archives, I use everything.” Most of the respondents (95%) also appreciate the ability to search and find their family through these platforms from the comfort of their home. Nearly all respondents (98%) appreciated how these digital platforms allow them to do family history research when and where they please. However, this factor seemed more important for those who do family history more often than for those who partake once a year or less. There was general agreement across age groups about the advantages. Nevertheless, when asked about issues with using digital platforms for family history nearly a third of the respondents (and more among the younger groups) had issues with the fact that not everything was available online. This was particularly true for those who had a more sporadic family history activity for example with more activity during holidays. However, online access seems crucial for many, especially access to sources that are geographically distant, for example in a different part of the country if confined to your home due to disability or for conducting this hobby while working full time. For instance, one of our focus group participants mentioned how important the online access was for initiating his engagement in family history, making it possible to combine with regular work: “To me, [a public platform] was an important entrance to get started because running around and looking at parish records, visiting libraries, and so on was definitely a barrier. So as [the platform] popped up and you got the opportunity to do different searches, that was what really got me started.” Other participants stressed the importance of material being accessible online, saying that as family history is a hobby and archives are far away it would be unreasonable to spend a lot of time and money on travels, making the online material more important. However, while it is great to have so much data online the problem, with access to sources that are not, remains. The latter are often sources that provide enrichment of the family history.

### Preserving findings for the future: Information management

After, or simultaneously as they search, family historians secure the new information for later access. This indicates that the practice of family historians is not restricted to knowing more about the family but also deals with retaining, referring, and preserving that new knowledge, making it theirs in some way. In the current buffet of available digital platforms, one could look up information as needed, as we do on Wikipedia, without needing to store the information locally. However, it is precisely the preservation and incorporation of these new sources of information into existing narratives (their own family history) that may be the hallmark of family history.

So, we questioned the practicalities behind that task: how they extract information from digital platforms and use it to further their family history activities. We were interested in knowing how they used this information or data and how they shared it with family history peers and with their own family members. Nearly all (96%) of the respondents said that they saved key information such as names, dates, and places. We already know that gathering these types of information is an important step in creating the scaffolding of the family tree on which to fasten a richer storyline. Thus, it is not surprising that they are also stored by nearly all the respondents.

While some platforms offer the option to store all findings with them it is very common that family historians extract that information out of the platforms while keeping track of the source metadata. Many respondents saved references to sources such as IDs (77%), as well as the scanned images themselves (71%). However, when it came to saving links to the many sources that are available on various platforms, only about half of the respondents did this. This practice is more common among the younger and middle-aged respondents who were also more likely to save images. Furthermore, those who have worked in cultural institutions or within the church seem more likely to save links and see this as an advantage of using digital platforms. This could be due to their knowledge about certain platforms and trusting that these provide a stable link to the original source. Perhaps this also aligns with the change Willever-Farr et al (2012) found in co-operative activities amongst family historians from aiding each other with instructions on how to search historical sources toward providing a direct link to a particular record.

Practically all respondents organize and store the information they collect in some way. In practice there seems to be some variety. Just over half the respondents store the information in an app or program on their computer and just over a third use word processing software. Perhaps surprisingly, a third of the respondents use a notebook or loose pages to store information collected online. This occurs more among women and among younger and middle-aged family historians. Whereas the older and more experienced family historians are more likely to store information on their own website. Twenty years ago, Yakel (2004) noted that the internet was providing opportunities to set up websites to share family trees with living relatives. However, while that may be the case, it seems that there is still an assortment of both digital and analogue methods for storing information. One issue that did come up in the focus group interviews was the lack of easy interoperability between platforms. One respondent said: “I have looked at [a platform's] family tree service. But since you cannot import your own family tree [from a program on her computer] and 25,000 people-you don’t sit and input them once more-so I won’t be using that.”

If collecting and preserving new information is a key activity of the family historian, it cannot be isolated from the critical evaluation of the reliability of that information. In the study, family historians showed a great deal of awareness about misleading or even fake information. In the interviews, there was an understanding about how especially transcribed sources may contain mistakes and how some, especially large international search facilities, can provide a great number of irrelevant results, particularly on the more common Danish names (e.g., Ane Hansen or Jens Nielsen). The earlier idea of family historians uncritically searching for that special connection to grand historical figures, or a ruling class (Redmann 1993) was nowhere to be found. There was rather a more critical stance toward this as well as a focus on the multitude of more nuanced stories that form a part of a more diverse historical narrative (Barclay and Kofoed 2021). Moving away from the idea of one patrilineage to a family tree with many roots and branches reveals a more heterogeneous structure (Foucault 1977). One where identity and sense of inheritance is as complex as the many individual life-stories that came before (Nash 2002). In this rhizomatic structure, the importance of meticulous record keeping is a vital tool. Here the platforms that provide direct links to sources are appreciated. This is a practical solution to the sharing of digitized original historical records that support the family tree.

### Being part of a community

Even when family historians sit alone in front of their computer, they are embedded in multiple sharing communities. On the one hand, there is the community established with their own kin: those they study and those still living who become the, sometimes reluctant, audience. On the other hand, there is the community of fellow family historians with whom they increasingly interact online through social media. Sometimes these communities merge into one as was the case with one interviewee: “In the beginning when my father had found something [about the family] I rolled my eyes and thought “now this again.” But since I have been doing it myself, I have become as smitten by it as he is.” Engaging with the community is an activity that both follows on from earlier phases and occurs simultaneously as they share experiences and seek and provide help. This engagement can happen across the different fora that some of the major platforms provide but especially in several Facebook groups.

Many respondents report using social media for keeping up with news and being a part of the community. Conversely sharing their own stories on social media is the least common form of engagement. Receiving help with reading handwriting, finding individuals in the historical sources, and generally finding information about historical subjects and places is, on the other hand, an important factor for those who use social media for their family history research. In the focus groups several mentioned the importance of giving and getting help in Facebook groups. “Fortunately, there are some, slightly older, family historians with good experience that can help those of us that cannot figure it out. I think that is what is great with these groups on Facebook. […] It usually does not take more than 20 s before someone comes up with an answer.” “And it is around the clock. It is amazing there are really some dedicated people behind that group, I really think so.”

They also emphasize the importance of helping others: “If people ask about something they should get an answer. If you can help then I think, well, it does not cost you anything.” There is a sense of positive reciprocity in this idea of helping others which relates to Fulton’s (2009a) findings. As one respondent put it: “We all have the same problems, I mean if it is not me today, then there is someone else tomorrow and if I got help yesterday then I might as well help, so I have also helped people in those family history groups. What you get you might as well pass on.” Additionally, the value of these exchanges is not restricted to the person asking for help. Many tips and ideas for new sources to search can be found by following the questions and answers of others in these communities.

Aiding your own family history by supporting others is furthermore related to the continuing and long-lasting crowdsourcing projects that form the basis of the Danish free access to census records. By transcribing the census of one parish which you have a particular interest in you are both directly helping yourself and indirectly the others who come along later. You are also contributing to a source where other people’s efforts are helping you. Even so, only a quarter of the respondents said that they did any type of heritage related volunteering.

In the focus group, it also became clear that the more ad hoc translation and transcription assignments that are possible to engage with through newer platforms like Danish Family Search suit some family historians better than the rather time-consuming transcription of a whole parish in the older census transcription project hosted by the Danish National Archives.

The relatively low number of participants in these crowdsourcing projects follows a pattern where a few do a large part of the work and many do a little bit each, which accumulates (Zeeland and Gronemann 2019). Therefore, you need both types of engagement. From this perspective, there is value in a transcription platform where you can contribute both a lot and a little. We also observed a sense of loyalty to older platforms that had served well in the past. However, personal preferences in communication and information behavior seems to triumph over loyalty and experience with a platform overall. For example, focus group interviewees preferred Facebook groups over the more traditional web forum structure for few other reasons than the speed at which questions were being answered. Meanwhile, other family historians might prefer the slower pace of the web forum where posts and answers can easily be searched and added to after many years.

## Conclusion

With an increased amount of archival material being made available online and an ever-growing family history community, this paper’s aim has been to further an understanding of family historians and their usage of digital platforms. Two main outcomes can be highlighted from our study of Danish family historians. First, a buffet model of engagement with digital platforms has been developed. It was simply the case that family historians approached online resources as a buffet where they pick and choose the available platforms that were most relevant for their task. They use a variety of platforms mixing the use of public, non-profit, for-profit, and social media platforms interchangeably. Platform usage is dependent on a variety of aspects such as the specific purpose, preferences, advertising, and some form of serendipity. Even though loyalties sometimes played a part, family historians are in general not devoted to particular platforms. They may have preferences but will switch if it serves their purposes and here both the ability to search through resources as well as access to original sources in a user-friendly manner is important. Furthermore, family historians use social media to help each other by providing links to original sources which is an issue with some of the older platforms which do not provide direct links to individual sources. Moreover, the buffet model includes four phases in the family historians’ work: building the skeleton of a family tree with key-information, enriching the data, information management, and participation in the wider community of family historians. These phases take place in a cyclical manner which can be both sequential and simultaneous. These different phases and how family historians move back and forth between these activities and the various platforms added to the analogy of a buffet.

That leads to our second outcome which is that family history tends to develop into a life-long activity. In fact, 60% of the respondents to our questionnaire had been active for more than 10 years. Even so, for some of the participants in our study it started as a discrete one-time project with a specific event, a spark, creating a wish for a family history. With time, it then evolved into what has been called a serious leisure career, meaning that they find deep meaning in their family history work while also building up a great deal of expertise in this field. It is noticeable that the longer they have worked with family history, the more interested they become in accessing other types of material that can enrich their stories, material that sometimes is less accessible online.

We introduced this study by mentioning the under-studied field of family historians. The need for a more comprehensive understanding of this group and their online practices across different cultural contexts becomes clear considering the current focus on engagement with digital platforms and democratic participation within the heritage sector. Thus, we want to offer archives and other heritage institutions a clear take-away from this research. Based on the outcomes we would suggest that these institutions focus their energy and resources on providing access to source-material that can enrich the family history narrative. We suggest that they do so by supporting family history research into their unique collections. Whether this is local photographs or knowledge about local buildings or more national collections about immigration, politics, or occupations. There are already successful platforms available for accessing basic key-information for community-building and for information management. However, collections providing enrichment of the family history are difficult to access in their current, often analog, form and require a great deal of expertise. Finally, experienced family historians seeking sources to enrich their family narrative hold the potential for rich and equally beneficial collaborations between institutions and an interested public.
